# Prospective association between plasma amino acids and three Multimorbidity patterns in older adults

**DOI:** 10.1038/s41598-025-06683-6

**Published:** 2025-07-01

**Authors:** Grace Velapatiño-Gamarra, Alberto Lana, Humberto Yévenes-Briones, Juan Cárdenas-Valladolid, José R. Banegas, Fernando Rodríguez-Artalejo, Esther López-García, Francisco Félix Caballero

**Affiliations:** 1 Department of Preventive Medicine and Public Health, Universidad Autónoma de Madrid, Madrid, Spain; 2 Department of Medicine, Universidad de Oviedo/ISPA, Oviedo, Spain; 3 CIBER of Epidemiology and Public Health, Instituto de Salud Carlos III, Madrid, Spain; 4 Dirección General de Investigación y Docencia, Consejería de Sanidad de la Comunidad de Madrid, Madrid, Spain; 5 Universidad Alfonso X El Sabio, Villanueva de la Cañada, Spain; 6 IMDEA-Food Institute. CEI UAM+CSIC, Madrid, Spain; 7 Department of Preventive Medicine and Public Health, School of Medicine, Universidad Autónoma de Madrid, C/Arzobispo Morcillo, 4, Madrid, 28029 Spain

**Keywords:** Amino acids, Musculoskeletal and mental Multimorbidity pattern, Cardiometabolic Multimorbidity pattern, Cardiopulmonary Multimorbidity pattern, Risk markers, Metabolomics, Biomarkers, Diseases

## Abstract

The role of metabolomic profiling on different multimorbidity patterns remains unknown. This study aims to assess the prospective relationship between plasma concentrations of amino acids and three different multimorbidity patterns: musculoskeletal and mental multimorbidity, cardiometabolic multimorbidity, and cardiopulmonary multimorbidity. The study comprised a total of 1488 older adults from the Seniors-ENRICA 2 Spanish cohort. Plasma concentrations of alanine, glutamine, glycine, histidine, branched-chain amino acids, and aromatic amino acids were measured. Generalized estimating equation models were used to assess the prospective association between amino acids and multimorbidity patterns. Higher plasma concentrations of branched-chain amino acids as leucine [Odds Ratio (OR) per 1-SD increment = 1.05, 95% confidence interval (CI) = 1.01–1.08)], isoleucine (OR = 1.05, 95% CI = 1.01–1.08), and valine (OR = 1.04, 95% CI = 1.01–1.08) were related to cardiometabolic multimorbidity, while lower concentrations of glycine (OR = 0.95, 95% CI = 0.91–0.99) and tyrosine (OR = 0.96, 95% CI = 0.93–0.98) were also associated with the same multimorbidity pattern. On the other hand, higher plasma concentrations of glutamine (OR = 1.18, 95% CI = 1.03–1.34) were related to cardiopulmonary multimorbidity. In conclusion, branched-chain amino acids may serve as risk markers of cardiometabolic multimorbidity in older adults. Plasma concentrations of other amino acid species such as glycine, tyrosine, and glutamine could also help to identify cardiometabolic and cardiopulmonary multimorbidity patterns.

**Supplementary Information:**

The online version contains supplementary material available at 10.1038/s41598-025-06683-6.

## Introduction

The world population is aging, and the proportion of people aged 60 years and over is expected to nearly double by 2050^1^. One of the reasons is the increase in life expectancy. However, since old age is the strongest risk factor for most chronic diseases, in many cases, these additional years are lived in poor health^2^. Chronic diseases involve a prolonged duration and either (a) residual disability or worsening quality of life or (b) a long period of care, treatment, or rehabilitation^3^. Although multimorbidity is generally defined as the coexistence of two or more chronic diseases, which is a useful approach for providing prevalence estimates and assessing its incidence in longitudinal studies, quantitative ways of measuring multimorbidity have been also proposed: one of them based on a simple count from a list of chronic diseases^4^, and another one introducing a multimorbidity weighted index, based on the impact of chronic diseases on physical health-related quality of life^5^ and physical functioning^6^. Furthermore, the nonrandom associations between chronic diseases are called multimorbidity patterns^7^, based on a previous proposal of categorical classification^8^. These nonrandom associations could suggest potential causal shared mechanisms.

Mechanisms underlying the development of multimorbidity patterns are unclear but there is an increased attempt to explain them. Sociodemographic characteristics as older age, lower socioeconomic status and female sex have been found to be related to multimorbidity^9^. On the other hand, lifestyle factors as smoking status, low physical activity, non-optimal sleep duration, and low diet quality have been associated with different multimorbidity patterns in older adults^10^.

Metabolomic profiles contribute to different pathways in the aging process^11^, and some efforts have been done on determining the prospective association between metabolites and multimorbidity^6,12–14^. Specifically, amino acids are metabolites associated with aging-related diseases^12,15^ and risk of disability^16^. Moreover, after considering different multimorbidity patterns in a cross-sectional study, a differential correlation was found between several metabolomic biomarkers and the types of multimorbidity patterns considered^17^.

Therefore, this research was focused on assessing a set of plasma amino acids and three multimorbidity patterns previously identified in older adults^10^. These multimorbidity patterns have been found to be differentially associated with social determinants of health and lifestyle behaviors. Accurate data on physician-diagnosed diseases were available based on primary care electronic clinical records and a wide set of chronic diseases has been used to characterize the studied multimorbidity patterns: musculoskeletal diseases and mental disorders, cardiometabolic diseases, and cardiopulmonary diseases. The study aims were: (1) To assess the prospective association between plasma amino acid concentrations and three different multimorbidity patterns, during a two-year follow-up period; and (2) To assess the risk of incidence of these multimorbidity patterns associated to baseline plasma amino acid concentrations, during a four-year follow-up period.

## Discussion

While some studies have investigated the association between amino acids concentrations and single diseases as diabetes^18^ or cardiovascular disease^19^, the present research explores the prospective relationship between plasma concentrations of a set of nine amino acid species (including BCAAs and AAAs) and three different multimorbidity patterns: musculoskeletal diseases and mental disorders, cardiometabolic diseases, and cardiopulmonary diseases. Although previous research has been conducted on concordant comorbid conditions which may have shared care goals and risks management^20,21^, there is a need of identifying determinants of disease clustering, considering different groups of chronic conditions which can be correlated among them.

We found that higher concentrations of BCAAs (isoleucine, leucine, and valine) and lower concentrations of glycine and tyrosine were prospectively associated with higher likelihood of cardiometabolic multimorbidity. This pattern included several chronic diseases such as diabetes, dyslipidemia, hypertension, and obesity.

BCAAs have been previously proposed as biomarkers of cardiometabolic diseases^22^. Cross-sectional associations were found in a Japanese population between elevated plasma BCAA levels and risk of lifestyle-related diseases as diabetes, dyslipidemia, hypertension, and obesity^23^. Also, higher plasma concentrations of BCAAs, specifically isoleucine and valine, were prospectively associated with higher levels of general multimorbidity (measured as a continuous variable) in the Seniors-ENRICA 2 Spanish cohort^13^, the same cohort which has been considered for the study aims of identifying metabolomic determinants of three different types of multimorbidity in older adults. Paradoxically, a higher risk of all-cause mortality and major cardiovascular endpoints (defined as having stroke, heart attack, heart failure, or revascularization) was found in a sample of 918 Australian men aged 70 years and older with low concentrations of BCAAs, although the association for major cardiovascular endpoints was not significant when adjusting for age and frailty^24^. These discoveries could suggest shared pathways among the diseases clustered in this pattern: the molecules involved in the catabolism of BCAAs, as well as the BCAAs themselves, would be affecting these cardiometabolic diseases. A recent review has concluded that elevated circulating BCAAs modulate various biochemical pathways to potentially affect cardiometabolic diseases, while by promoting the transport of fatty acids or influencing over the Mammalian Target of Rapamycin Complex 1 (mTORC1) and insulin resistance, these diseases would generate positive feedback that would lead to the elevation of BCAAs^25^. A previous study has suggested that plasma concentrations of BCAAs could be used as a predictor for future metabolic diseases among obese and overweight people^26^, although further studies of mechanisms by which BCAAs and related metabolites affect cardiometabolic disease pathogenesis are still needed^22^.

In line with our results on the association between low plasma glycine levels and cardiometabolic multimorbidity pattern, a narrative review identified lower plasma glycine levels in obesity, diabetes, and non-alcoholic fatty liver disease (NAFLD)^27^. In addition, an association between NAFLD and dyslipidemia and hypertension has also been reported^28^. However, while our results showed an inverse relationship between tyrosine and cardiometabolic multimorbidity pattern, a previous case-control study in middle-aged adults found that higher plasma tyrosine levels were associated with a higher risk of diabetes^29^.

Higher concentrations of glutamine were associated with cardiopulmonary multimorbidity pattern. A clinical trial evaluating daily supplementation of L-glutamine in atrial fibrillation patients, a chronic disease included in our cardiopulmonary multimorbidity pattern, found that increased plasma concentrations of glutamine could have cardioprotective effects that may attenuate atrial fibrillation episodes^30^. Another study conducted in the Seniors-ENRICA 2 Spanish cohort showed that higher plasma concentrations of glutamine were prospectively related to higher levels of general multimorbidity^13^, after considering a set of 60 chronic conditions without distinguishing between different types of multimorbidity. Furthermore, a case-control study found that higher plasma concentrations of glutamine were related to increased risk of disability in older adults^16^. Glutamine is the most abundant amino acid in the human body^31^, but excessively high concentrations of this metabolite could be a predictive marker of frailty in older adults^32^. While higher plasma concentrations of glutamine have been found to be associated with higher levels of general multimorbidity^13^, the present study has identified a specific relationship of higher plasma concentrations in this amino acid with the presence of cardiopulmonary multimorbidity; this significant association has not been found with the remaining two multimorbidity patterns considered.

An additional analysis was conducted in our study, exploring the amino acid profiling associated with incident multimorbidity patterns. A significant association was found between higher baseline plasma concentrations of isoleucine and risk of incidence of cardiometabolic and cardiopulmonary multimorbidity patterns. Circulating plasma BCAAs have been found to be positively associated with incident cardiovascular disease in an US women cohort^33^. Specifically, elevated concentrations of circulating isoleucine have been associated with increased risk of cardiovascular disease in a systematic review with meta-analysis of non-prospective and prospective clinical studies^34^. According to the results found in the GEE models, higher plasma concentrations of isoleucine were prospectively associated with cardiometabolic multimorbidity pattern, but this association was not found for cardiopulmonary multimorbidity pattern. While the prospective association between amino acids and multimorbidity patterns was assessed using information available from a two-year follow up period (2017–2019), information on physician-based diagnoses was updated in December 2021. Therefore, a four-year follow-up period was considered for the incidence analysis; however, our results should be interpreted with caution given the small number of incident cases in the corresponding multimorbidity patterns.

To the best of our knowledge, we provide the first evidence on the prospective association between the amino acids considered and three different multimorbidity patterns. While a previous study used data from the Seniors-ENRICA 2 cohort to assess the relationship between amino acids profiling and multimorbidity^13^, the present research shows original findings, after identifying how different amino acid species can be prospectively associated with different multimorbidity patterns. While the concept of multimorbidity refers traditionally to the simultaneous presence of two or more chronic conditions, multimorbidity patterns are specific clusters or groupings of these chronic conditions, reflecting common co-occurrences and potential shared underlying factors^35,36^.

This is a population-based cohort study where socio-demographic, socioeconomic and lifestyle factors have been well characterized, and the study information has been linked to clinical records morbidity data. Chronic diseases used to define each multimorbidity pattern have been adequately coded, using the ICPC-2 in primary care settings and the ICD-10 in hospital settings. The GEE models built for exploring the prospective associations considered have been adjusted for a wide range of potential confounders. Moreover, these models considered the longitudinal information of the study and the within-subject correlation across the two phases (baseline and the follow-up phase conducted in 2019) in which plasma amino acid concentrations were measured.

Among the potential limitations of this study, some amino acid species as tryptophan have not been assessed and further research should disentangle the prospective association between other amino acid species and different multimorbidity patterns. The lab where plasma samples were sent to be analyzed provides targeted metabolomics and measures nine amino acid concentrations, which we have considered for our research. Future research lines could explore the mutual effects of all protein amino acids on multimorbidity patterns. This said, the set of amino acids used in this study includes BCAAs and AAAs. Another limitation is that the GEE models for repeated measures were not adjusted for diet quality since this variable was not measured in the follow-up phase. Adjusting for diet-related variables could be relevant since some of the amino acids evaluated are essentials, which means that they depend on the nutrient intake; however, plasma samples were collected under 12-h fasting, mitigating the absence of this variable among the covariates. Regarding the relationship between amino acid profiling and incident multimorbidity patterns, this should be interpreted with caution due to the relatively short follow-up period considered and the relatively small number of incident cases found for the three different multimorbidity patterns. Moreover, according to the research design, it cannot be ruled out that the findings were attributed to reverse causation.

In conclusion, this study found a significant prospective association between higher concentrations of BCAAs and cardiometabolic multimorbidity pattern. In the same line, higher concentrations of glutamine and isoleucine were significantly associated with the presence and incidence of cardiopulmonary multimorbidity pattern, respectively. On the other hand, higher concentrations of glycine and tyrosine were related to lower levels of cardiopulmonary multimorbidity pattern. The role of other amino acid species, as tryptophan, on aging-related diseases as multimorbidity patterns should be assessed in further studies. Amino acids could play a role in regulating aging-related diseases and our findings can help to identify risk markers differentially associated with different multimorbidity patterns in older adults.

## Results

A total of 1488 people were included in the study, of whom 738 (49.6%) were women and 750 (50.4%) were men. Baseline characteristics are reported in Table 1. The mean age was 71.3 (SD = 4.2) years. The most common educational attainment was primary education or less, present in 875 participants (58.8%). There were significant differences between men and women in the prevalence of two multimorbidity patterns (musculoskeletal diseases and mental disorders, and cardiopulmonary diseases), with a higher prevalence in women (71.7% vs. 25.2%) in the first pattern, and a higher prevalence in men (6.7% vs. 3.3%) in the second one. Significant differences by sex were not found for cardiometabolic multimorbidity. Supplementary Table S1 shows the plasma concentrations of the nine amino acids included in the study. There were significant differences by sex in the plasma concentrations of glycine, histidine, isoleucine, leucine, valine, and phenylalanine.

**Table 1:** Baseline socio-demographics and clinical characteristics of the study sample (*N* = 1488). Mean ± Standard deviation.

	Overall *N* = 1488	Men *n* = 750	Women *n* = 738	P*
Age, years	71.3 ± 4.2	71.0 ± 4.0	71.5 ± 4.3	0.014
Educational attainment, n (%)				
Primary education or less	875 (58.8)	381 (50.8)	494 (67.0)	< 0.001
Secondary school	279 (18.8)	153 (20.4)	126 (17.1)	
University studies	333 (22.4)	216 (28.8)	117 (15.9)	
Householder’s occupational category, n (%)				
Professionals and managers	169 (11.4)	72 (9.6)	97 (13.2)	< 0.001
Lower non-manual workers	375 (25.2)	168 (22.4)	207 (28.1)	
Skilled manual workers	551 (37.1)	278 (37.0)	273 (37.1)	
Unskilled manual workers	390 (26.3)	231 (30.8)	159 (21.6)	
Body mass index, kg/m^2^	27.6 ± 4.3	27.6 ± 3.8	27.5 ± 4.7	0.61
Never smokers, n (%)	722 (48.5)	506 (67.5)	216 (29.3)	< 0.001
Optimal sleep duration, n (%)	783 (53.0)	431 (57.7)	352 (48.2)	< 0.001
AHEI-2010 score	63.2 ± 9.6	63.7 ± 9.5	62.6 ± 9.7	0.025
Musculoskeletal and mental multimorbidity pattern, n (%)	718 (48.3)	189 (25.2)	529 (71.7)	< 0.001
Cardiometabolic multimorbidity pattern, n (%)	706 (47.5)	343 (45.8)	363 (49.2)	0.19
Cardiopulmonary multimorbidity pattern, n (%)	74 (5.0)	50 (6.7)	24 (3.3)	0.002

Note: SD = Standard deviation; AHEI = Alternate Healthy Eating Index; *Unpaired t-test was used for comparing means in quantitative variables by sex, while chi-square test was used for comparing percentages in categorical variables by sex.

In GEE models adjusted for potential confounders, significant associations were not found between any of the plasma amino acids and the musculoskeletal and mental multimorbidity pattern (Table 2). Lower plasma concentrations of glycine were associated with cardiometabolic multimorbidity pattern (OR per 1-SD increment = 0.95, 95% CI = 0.91–0.99; *p* = 0.046). Significant associations were also found between the BCAAs and cardiometabolic multimorbidity pattern: higher concentrations of isoleucine (OR = 1.05, 95% CI = 1.01–1.08; *p* = 0.006), leucine (OR = 1.05, 95% CI = 1.01–1.08; *p* = 0.004), and valine (OR = 1.04, 95% CI = 1.01–1.08; *p* = 0.005) were prospectively associated with the presence of cardiometabolic multimorbidity pattern, as can be observed in Table 3. Regarding AAAs, lower concentrations of tyrosine were associated with cardiometabolic multimorbidity pattern (OR = 0.96, 95% CI = 0.93–0.98; *p* = 0.002) (Table 3). Finally, higher concentrations of glutamine were significantly associated with cardiopulmonary multimorbidity pattern (OR = 1.18, 95% CI = 1.03–1.34; *p* = 0.016) (Table 4). However, significant associations between BCAAs or AAAs and cardiopulmonary multimorbidity pattern were not observed. When including an interaction term for sex and the corresponding amino acid in those models who reflected a significant association between the amino acid and the corresponding multimorbidity pattern, the interaction term was not found to be significant, suggesting no differential relationships by sex.

**Table 2:** Odds ratios (95% confidence interval) per one standard deviation increase for the prospective association between plasma amino acid concentrations (mmol/L) and musculoskeletal and mental Multimorbidity pattern.

	Model 1		Model 2	
	Odds ratio per 1-SD increase (95% CI)	*p*	Odds ratio per 1-SD increase (95% CI)	*p*
Alanine	0.98 (0.94–1.02)	0.30	0.97 (0.93–1.01)	0.13
Glutamine	1.00 (0.95–1.05)	0.97	1.00 (0.95–1.05)	0.85
Glycine	0.96 (0.88–1.05)	0.43	0.95 (0.87–1.03)	0.20
Histidine	0.99 (0.96–1.03)	0.67	0.99 (0.95–1.02)	0.43
Branched-chain amino acids				
Isoleucine	0.98 (0.94–1.02)	0.29	0.97 (0.93–1.01)	0.19
Leucine	0.97 (0.93–1.02)	0.20	0.96 (0.92–1.01)	0.11
Valine	0.98 (0.94–1.03)	0.41	0.97 (0.93–1.02)	0.25
Aromatic amino acids				
Phenylalanine	1.00 (0.95–1.05)	0.94	0.99 (0.94–1.04)	0.69
Tyrosine	1.01 (0.96–1.06)	0.61	1.00 (0.95–1.06)	0.86

Note: Separated generalized estimating equation models were run for each of the nine amino acids considered; Model 1 was adjusted for sex, age, educational level attained, and householder’s occupational category; Model 2 was additionally adjusted for lifestyle behaviors (physical activity, body mass index, optimal sleep duration, and tobacco consumption).

**Table 3:** Odds ratios (95% confidence interval) per one standard deviation increase for the prospective association between plasma amino acid concentrations (mmol/L) and cardiometabolic Multimorbidity pattern.

	Model 1		Model 2	
	Odds ratio per 1-SD increase (95% CI)	*p*	Odds ratio per 1-SD increase (95% CI)	*p*
Alanine	1.03 (1.00–1.06)	0.037	1.02 (0.99–1.05)	0.19
Glutamine	0.99 (0.96–1.03)	0.75	1.00 (0.96–1.04)	0.96
Glycine	0.94 (0.90–0.98)	0.003	0.95 (0.91–0.99)	0.046
Histidine	1.00 (0.98–1.02)	0.98	1.00 (0.98–1.02)	0.98
Branched-chain amino acids				
Isoleucine	1.05 (1.02–1.08)	0.001	1.05 (1.01–1.08)	0.006
Leucine	1.05 (1.02–1.08)	0.001	1.05 (1.01–1.08)	0.004
Valine	1.05 (1.02–1.08)	< 0.001	1.04 (1.01–1.08)	0.005
Aromatic amino acids				
Phenylalanine	1.01 (0.99–1.04)	0.28	1.01 (0.98–1.04)	0.54
Tyrosine	0.98 (0.95–1.01)	0.08	0.96 (0.93–0.98)	0.002

Note: Separated generalized estimating equation models were run for each of the nine amino acids considered; Model 1 was adjusted for sex, age, educational level attained, and householder’s occupational category; Model 2 was additionally adjusted for lifestyle behaviors (physical activity, body mass index, optimal sleep duration, and tobacco consumption).

**Table 4:** Odds ratios (95% confidence interval) per one standard deviation increase for the prospective association between plasma amino acid concentrations (mmol/L) and cardiopulmonary Multimorbidity pattern.

	Model 1		Model 2	
	Odds ratio per 1-SD increase (95% CI)	*p*	Odds ratio per 1-SD increase (95% CI)	*p*
Alanine	1.04 (0.94–1.13)	0.46	1.01 (0.91–1.12)	0.89
Glutamine	1.13 (0.99–1.29)	0.07	1.18 (1.03–1.34)	0.016
Glycine	0.97 (0.83–1.13)	0.73	0.99 (0.84–1.15)	0.87
Histidine	0.92 (0.84–1.01)	0.10	0.92 (0.83–1.01)	0.08
Branched-chain amino acids				
Isoleucine	1.03 (0.93–1.15)	0.53	1.00 (0.90–1.12)	0.93
Leucine	0.98 (0.88–1.10)	0.74	0.95 (0.84–1.06)	0.35
Valine	1.00 (0.89–1.13)	0.97	0.96 (0.85–1.09)	0.55
Aromatic amino acids				
Phenylalanine	0.97 (0.85–1.11)	0.67	0.94 (0.82–1.08)	0.37
Tyrosine	0.96 (0.83–1.10)	0.53	0.94 (0.81–1.08)	0.36

Note: Separated generalized estimating equation models were run for each of the nine amino acids considered; Model 1 was adjusted for sex, age, educational level attained, and householder’s occupational category; Model 2 was additionally adjusted for lifestyle behaviors (physical activity, body mass index, optimal sleep duration, and tobacco consumption).

Logistic regression models were also conducted to assess separately the association between each baseline plasma amino acid concentration and the incidence of each multimorbidity pattern, and higher concentrations of isoleucine were found to be associated with risk of incidence of cardiometabolic (OR = 1.37, 95% CI = 1.02–1.84; *p* = 0.037) and cardiopulmonary (OR = 1.28, 95% CI = 1.01–1.64; *p* = 0.046) multimorbidity pattern (Table 5).

**Table 5:** Odds ratios (95% confidence interval) per one standard deviation increase for assessing the relationship between baseline plasma amino acid concentrations (mmol/L) and four-year incidence of Multimorbidity patterns.

	Musculoskeletal and mental multimorbidity pattern		Cardiometabolic multimorbidity pattern		Cardiopulmonary multimorbidity pattern	
Total/Incident cases	**769/71**		**781/73**		**1413/43**	
	**Odds ratio per 1-SD increase (95% CI)**	***p***	**Odds ratio per 1-SD increase (95% CI)**	***p***	**Odds ratio per 1-SD increase (95% CI)**	***p***
Alanine	1.02 (0.79–1.33)	0.87	1.28 (0.98–1.67)	0.07	1.13 (0.83–1.54)	0.45
Glutamine	1.06 (0.81–1.38)	0.67	1.09 (0.85–1.41)	0.50	1.03 (0.75–1.42)	0.85
Glycine	1.07 (0.81–1.42)	0.62	1.05 (0.81–1.36)	0.72	1.01 (0.72–1.43)	0.95
Histidine	0.82 (0.63–1.07)	0.14	0.81 (0.62–1.05)	0.11	1.14 (0.83–1.56)	0.43
Branched-chain amino acids						
Isoleucine	0.93 (0.71–1.20)	0.56	1.37 (1.02–1.84)	0.037	1.28 (1.01–1.64)	0.046
Leucine	0.97 (0.75–1.26)	0.82	1.31 (0.97–1.77)	0.08	1.14 (0.85–1.54)	0.38
Valine	1.00 (0.77–1.29)	0.99	1.27 (0.95–1.69)	0.10	1.10 (0.80–1.51)	0.55
Aromatic amino acids						
Phenylalanine	1.03 (0.83–1.28)	0.76	1.04 (0.83–1.30)	0.74	1.10 (0.85–1.42)	0.49
Tyrosine	1.09 (0.85–1.39)	0.52	1.21 (0.93–1.57)	0.16	1.15 (0.87–1.51)	0.32

Note: Separated logistic regression models were run for each of the nine amino acids considered; each model was adjusted for sex, age, educational level attained, householder’s occupational category, and lifestyle behaviors (physical activity, body mass index, optimal sleep duration, diet quality and tobacco consumption). For each multimorbidity pattern, prevalent cases at baseline were excluded from the analysis.

## Methods

### Study design

The study sample comprises non-institutionalized 1488 subjects aged ≥ 65 years from the Seniors-ENRICA 2 cohort^37,38^. Participants in this prospective study lived in the city of Madrid (Spain) and four nearby towns, and held a national health care; they were recruited between 2015 and 2017 by sex- and district-stratified random sampling. A follow-up phase was conducted in 2019. The instruments, procedures used for data collection, and study design are similar to the methods described for the Seniors-ENRICA 1 cohort^39^. Sociodemographic characteristics, lifestyle behaviors and health-related conditions were collected by computer-assisted telephone interviews, and two home visits were subsequently conducted to perform a physical examination and obtain biological samples. Twelve-hour fasting blood samples were collected by trained nurses at the participants’ home under standardized conditions. The study was approved by the Clinical Research Ethics Committee of ‘La Paz’ University Hospital in Madrid (PI-1793, PI-3554) and the study participants gave written informed consent to be involved. All methods were performed in accordance with the relevant guidelines and regulations.

The Research Central Commission of Madrid Regional Health Service (SERMAS), Primary Health Care, provided permission to access the medical history data set. The quality of the primary care electronic clinical record has previously been validated for research purposes, and the data set has been widely used to study the epidemiology of cardiovascular risk factors in the study population^40^.

### Sociodemographic and socioeconomic status

Participants reported their age and sex. Socioeconomic status was measured based on educational attainment and the latest householder´s occupational category. Educational attainment was classified into primary education or less, secondary school and university studies, while householder´s occupational category was classified into professionals and managers, lower non-manual workers, skilled manual workers, and unskilled manual workers^41^.

### Lifestyle behaviors

Body mass index (BMI), tobacco consumption, physical activity, sleep duration, and diet quality were assessed. BMI was calculated as weight (kg) divided by squared height (m), both measured in standardized conditions. Tobacco consumption was categorized as “never smokers” and “current or former smokers”. Physical activity in metabolic equivalent tasks (METs-hours/week) was assessed with the validated questionnaire developed in the EPIC cohort study in Spain^42^ and participants were asked for the number of hours that they spent in a typical week during the last year on each of the following activities: walking, cycling, gardening, do-it-yourself activities at home, playing sports (running, fitness, aerobics, swimming, soccer, tennis, etc.), and climbing stairs. Tertiles of the distribution of BMI and physical activity were derived. Sleep duration was self-reported and measured as the number of hours slept per day (h/d); optimal sleep duration was defined as 7–8 h per day^43^.

Habitual food consumption was collected with a validated computerized diet history^44^. Information on diet quality was only available at baseline and was estimated by the Alternate Healthy Eating Index-2010 (AHEI-2010)^45^, which is based on a set of 11 foods and nutrients predictive of chronic disease risk. For each food and nutrient, a value of 10 was assigned to low/no intakes of unhealthy foods and nutrients, whereas a value of 0 was assigned to higher consumption of unhealthy dietary components, with intakes between the minimum and maximum levels being proportionally scored. Scores were added up for obtaining a global score in the AHEI-2010.

### Amino acid species

Blood samples were collected in ethylene diamine tetraacetic acid tubes, centrifuged at 3000 rpm for 10 min and aliquoted in the following 24 h from collection. The plasma samples were stored at − 70 °C at the Universidad Autónoma de Madrid Biobank until their processing at the Nightingale Health laboratory (Nightingale Health Ltd, Helsinki, Finland). Sample volumes of 350 µL were used for the analysis. High-throughput proton nuclear magnetic resonance (NMR)^46,47^ was used to quantify amino acid concentrations, and the inter-batch coefficient of variation ranged between 2.5% and 9.9% across the amino acids analyzed. Plasma amino acid concentrations were measured at baseline and 2019 for nine amino acid species: alanine, glutamine, glycine, histidine, isoleucine, leucine, valine, phenylalanine, and tyrosine. Isoleucine, leucine, and valine are branched-chain amino acids (BCAAs)^48^, while phenylalanine and tyrosine are aromatic amino acids (AAAs)^49^.

### Multimorbidity patterns

Participants’ information was linked to electronic health records; thus, physician-based diagnoses and their dates were available to characterize multimorbidity patterns at baseline (using diagnoses coded until December 2017), follow-up phase (diagnoses until December 2019), and 2021 (diagnoses until December 2021) for exploring the incidence of multimorbidity patterns in a four-year period. In all cases, chronic diseases were coded with the International Classification of Primary Care, 2nd Edition (ICPC-2), in primary care settings and an internationally derived operational measure^3^ was used for proposing the chronic conditions to be included in each multimorbidity pattern. We considered three multimorbidity patterns that were identified previously, by means of factor analysis, in the Seniors-ENRICA 2 cohort^10^. The musculoskeletal and mental multimorbidity pattern was defined as the presence of two or more conditions from the following list of eight musculoskeletal and mental chronic conditions: depression and mood diseases; migraine and facial pain; neurotic, stress-related and somatoform diseases; osteoarthritis and other degenerative joint diseases; osteoporosis; other musculoskeletal and joint diseases; other psychiatric and behavioral diseases; and thyroid diseases. The cardiometabolic multimorbidity pattern consisted of two or more of the following four metabolomic chronic conditions: diabetes; dyslipidemia; hypertension; and obesity. Finally, the cardiopulmonary multimorbidity pattern was defined as having two or more conditions among atrial fibrillation; cardiac valve disease; cerebrovascular disease; chronic obstructive pulmonary disease; ischemic heart disease; and peripheral vascular disease^10^. Chronic conditions considered in this research are exclusive of one of the multimorbidity patterns and cannot be included in more than one of the multimorbidity patterns identified.

### Statistical analysis

Baseline sociodemographic and clinical characteristics of the study sample were described. Potential differences by sex were analyzed by unpaired t-test for comparing means in quantitative variables and chi-square test for comparing percentages in categorical variables. Geometric means and 95% confidence intervals (CI) were calculated by sex at baseline for the raw amino acid concentrations. To deal with potential deviations from normal distributions, raw values of amino acids were log-transformed before comparing concentrations between men and women by means of unpaired t-tests.

Generalized estimating equation (GEE) models^50^ were used to assess the longitudinal association between plasma amino acid concentrations and the three types of multimorbidity assessed (musculoskeletal and mental multimorbidity, cardiometabolic multimorbidity, and cardiopulmonary multimorbidity), after considering the information available at baseline and 2019 for amino acid concentrations and the remaining covariates. GEE is a method for modeling longitudinal or clustered data and produces good estimates of model parameters when analyzing repeated measures. A logit link function and an exchangeable correlation structure were used to account for the binary nature of outcome variables and the correlation between repeated measures of amino acid concentrations in the same individual. The robust variance estimator was used to account for the within-subject correlation. Separated GEE models were run for each of the 9 amino acids measured. Each model was firstly adjusted for age, sex, educational level attained, and householder’s occupational category (model 1); and then was additionally adjusted for lifestyle behaviors, including physical activity, body mass index, optimal sleep duration, and tobacco consumption (model 2). In order to test potentially different relationships by sex, between plasma amino acids and multimorbidity patterns, an interaction term for sex and the corresponding amino acid was additionally included in those fully adjusted models who reflected a significant association between the amino acid and the multimorbidity pattern. As an additional analysis, logistic regression models were also conducted to assess the relationship between plasma amino acid concentrations and four-year incidence of multimorbidity patterns; thus, incident cases were defined for each multimorbidity pattern after excluding participants who had the corresponding multimorbidity pattern at baseline. These models were adjusted for age, sex, educational level attained, householder’s occupational category, and lifestyle behaviors (physical activity, body mass index, optimal sleep duration, diet quality, and tobacco consumption) at baseline. Since participants’ information was linked to electronic health records, physician-based diagnoses were available and updated in December 2021; therefore, this analysis was conducted over a four-year period.

Statistical analyses were conducted using Stata version 17.

## Electronic supplementary material

Below is the link to the electronic supplementary material.

Supplementary Material 1

## Data Availability

The datasets generated and analyzed during the current study are available from the corresponding author on reasonable request.
