# Supplementary material for: Prospective association between plasma amino acids and three Multimorbidity patterns in older adults
Source: Sci Rep. 2025 Jul 1;15:21668. doi: 10.1038/s41598-025-06683-6 (PMC12214811; doi:10.1038/s41598-025-06683-6)
Supplement: Supplementary file 1 — Supplementary Material 1 [file 41598_2025_6683_MOESM1_ESM.docx]

**Supplementary Table S1.** Geometric means (95% confidence intervals) of plasma amino acids concentrations(µmol/L) by sex at baseline.

|  | **Men** | **Women** | ***p*** |
| --- | --- | --- | --- |
| Alanine | 382.2 (376.2-388.3) | 374.6 (368.6-380.7) | 0.08 |
| Glutamine | 647.1 (641.9-652.4) | 640.6 (635.5-645.6) | 0.08 |
| Glycine | 168.0 (165.3-170.8) | 210.3 (205.5-215.2) | <0.001 |
| Histidine | 71.3 (70.7-72.0) | 67.8 (67.2-68.4) | <0.001 |
| Branched-chain amino acids |  |  |  |
| Isoleucine | 63.3 (62.3-64.4) | 51.4 (50.4-52.3) | <0.001 |
| Leucine | 139.2 (137.4-141.0) | 117.1 (115.4-118.7) | <0.001 |
| Valine | 258.6 (256.0-261.3) | 234.8 (232.1-237.5) | <0.001 |
| Aromatic amino acids |  |  |  |
| Phenylalanine | 59.1 (58.4-59.8) | 55.1 (54.4-55.8) | <0.001 |
| Tyrosine | 70.3 (69.5-71.1) | 69.6 (68.8-70.4) | 0.24 |

Note: *p*-values were obtained after applying an unpaired t-test over the log-transformed values.
